# Immunoglobulin A Vasculitis-Associated Ileoileal Intussusception in an Adult Male: Case Report

**DOI:** 10.5811/cpcem.63968

**Published:** 2026-08-06

**Authors:** Preet Sawhney, Patrick Frost, Peter Stueve, Eric Boccio

**Affiliations:** Mount Sinai Medical Center of Florida, Department of Emergency Medicine, Miami Beach, Florida

**Keywords:** IgA vasculitis, ileo-ileal intussusception, Henoch-Schönlein purpura, palpable purpura, case report

## Abstract

**Introduction:**

Immunoglobulin A (IgA) vasculitis, formerly known as Henoch–Schönlein purpura, is a small-vessel leukocytoclastic vasculitis caused by IgA immune complex deposition. While it is the most common systemic vasculitis in children, adult cases can present with more severe systemic manifestations. The classic clinical tetrad includes palpable purpura, arthralgia, abdominal pain, and renal involvement. Gastrointestinal symptoms, occurring in approximately two-thirds of cases, result from inflammation of small bowel vessels resulting in bowel wall edema and hemorrhage, which may serve as lead points for intussusception.

**Case Report:**

A 21-year-old male presented with two days of severe periumbilical abdominal pain, bilateral knee pain, and a nonblanching palpable purpuric rash on his lower extremities. Physical examination revealed a soft but tender abdomen. Lab results were remarkable for leukocytosis. Computed tomography (CT) of the abdomen and pelvis demonstrated small bowel wall thickening and ileoileal intussusception. The patient was initially consented for a partial ileal resection; however, an exploratory laparotomy failed to localize the telescoping segment, suggesting spontaneous resolution. He was observed for 24 hours and discharged with outpatient follow-up.

**Conclusion:**

Intussusception is the most common surgical complication of IgA vasculitis. In adults, this condition requires high clinical suspicion and prompt diagnostic imaging with ultrasonography or CT, as small bowel involvement may be inaccessible by contrast enema. This case underscores the importance of recognizing IgA vasculitis-associated intussusception as a critical and potentially self-limiting complication in the adult population.

## INTRODUCTION

Immunoglobulin A (IgA) vasculitis, formerly known as Henoch–Schönlein purpura, is a small-vessel leukocytoclastic vasculitis caused by IgA immune complex deposition. Although most common in children, adult cases may present with more severe systemic manifestations. The classic clinical presentation includes palpable purpura, abdominal pain, and arthralgias, with renal involvement occurring variably. Gastrointestinal symptoms result from immune complex-mediated inflammation of small bowel vessels resulting in bowel wall edema and hemorrhage, which may serve as lead points for intussusception.

## CASE REPORT

A 21-year-old male with no past medical or surgical history presented with two days of severe, intermittent periumbilical abdominal pain. Three days preceding the onset of abdominal pain, the patient reported having completed a five-day course of doxycycline for suspected atypical pneumonia. He reported associated bilateral knee pain and the development of a rash across the bilateral lower extremities. He denied fever, chills, nausea, vomiting, diarrhea, hematochezia, melena, urethritis, and penile discharge. The patient denied recent trauma and prior recreational drug use.

**Image 1:**
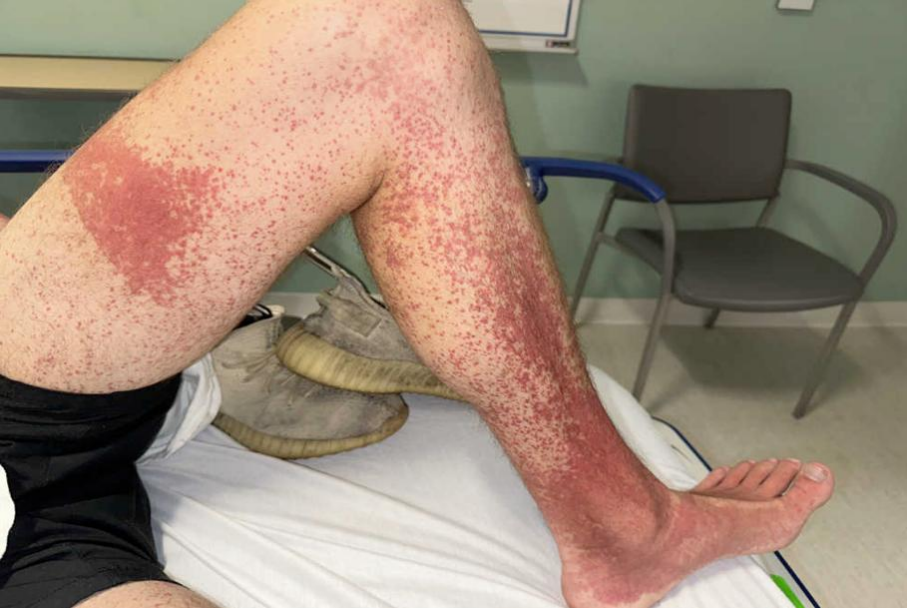
Red/purple palpable, nonblanching purpuric rash visualized on the left lower extremity in an adult male presenting with two days of periumbilical abdominal pain and bilateral knee pain.

**Image 2:**
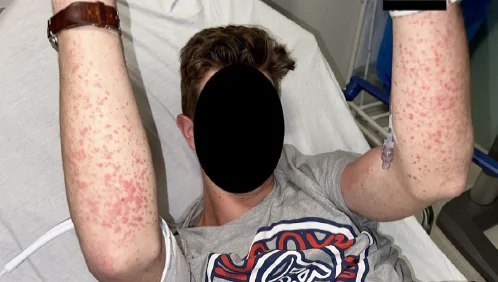
Red/pink and blanching flat discolorations and small raised bumps consistent with maculopapular eruption on the bilateral upper extremities in an adult male presenting with two days of periumbilical abdominal pain and bilateral knee pain.

Initial vital signs revealed the following: blood pressure, 143/71 millimeters of mercury; heart rate, 89 beats per minute; respiratory rate, 16 breaths per minute; peripheral capillary oxygen saturation, 99% on room air; and oral temperature, 36.9 degrees Celsius. The physical examination was remarkable for a soft and nondistended abdomen with maximal tenderness just inferior to the umbilicus. There was no rebound or guarding. Nonblanching palpable purpura were observed across the bilateral feet, ankles, and lower legs extending proximally (Image 1). A blanching maculopapular rash was also present across the bilateral upper extremities (Image 2).

**Image 3:**
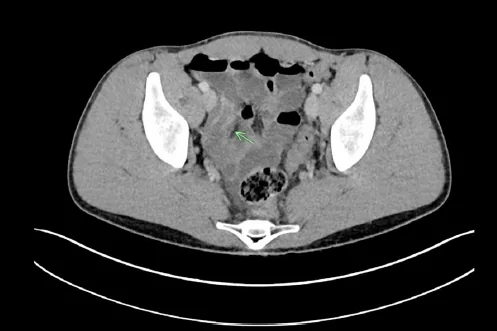
Computed tomography of the abdomen and pelvis demonstrating small bowel wall thickening with associated telescoping lead point consistent with ileoileal intussusception in a patient with IgA vasculitis (arrow).

A complete blood count was notable for a leukocytosis of 12.7 × 10^9^ cells/liter (reference range, 4.5–11 × 10^9^ cells/liter). The comprehensive metabolic panel, lipase, lactic acid, and urinalysis were within normal limits. Computed tomography (CT) of the abdomen and pelvis demonstrated small bowel wall thickening with associated ileoileal intussusception (Image 3).

Given the palpable nonthrombocytopenic purpura of the lower extremities, knee arthralgias, abdominal pain, and CT imaging demonstrating small bowel wall thickening and telescoping lead point, the diagnosis of IgA vasculitis-associated ileoileal intussusception was made. The patient received supportive care in the emergency department (ED), which included intravenous fluids and analgesics for abdominal pain. General surgery was consulted, and the patient was consented for a partial ileal resection. Exploratory laparotomy failed to localize the telescoping segment of bowel. The patient was observed for 24 hours and discharged with outpatient gastroenterology follow-up and strict return precautions.

*CPC-EM Capsule*

What do we already know about this clinical entity?

*Immunoglobulin A (IgA) vasculitis is a small-vessel vasculitis characterized by a tetrad of palpable purpura, arthralgia, abdominal pain, and renal involvement.*

What makes this presentation of disease reportable?

*This case features a rare adult presentation of IgA vasculitis-associated ileo-ileal intussusception discovered on computed tomography.*

What is the major learning point?

*Abdominal pain in adults with IgA vasculitis may represent small-bowel intussusception resulting from submucosal edema and hemorrhage acting as transient lead points.*

How might this improve emergency medicine practice?

*Clinicians should consider conservative management for stable cases and surgical intervention if signs of peritonitis or hemodynamic instability are present.*

## DISCUSSION

Immunoglobulin A vasculitis is the most common systemic vasculitis in childhood, with peak incidence at four to six years of age.^1^ This immune complex-mediated, small-vessel leukocytoclastic vasculitis is characterized by the deposition of IgA-dominant immune complexes in venules, capillaries, and arterioles, resulting in a classic tetrad of nonthrombocytopenic palpable purpura, arthritis or arthralgia, abdominal pain, and renal involvement.^2^ The disease often follows an upper respiratory tract infection. Gastrointestinal involvement occurs in approximately two-thirds of cases and represents a significant source of morbidity. The pathophysiology involves leukocytoclastic vasculitis of submucosal vessels, leading to bowel wall edema, hemorrhage, and potential ischemia. Abdominal symptoms may precede the characteristic purpuric rash in 14–36% of cases, potentially mimicking an acute surgical abdomen and complicating early diagnosis.^3^ While most gastrointestinal manifestations are self-limited, major complications develop in 1.3–13.6% of patients, with intussusception representing the most common surgical complication.^4^

Intussusception in IgA vasculitis differs from idiopathic intussusception in several key aspects. IgA-associated intussusception is confined to the small bowel in approximately 58% of cases, most commonly presenting as ileoileal or ileocolic intussusception.^5^ Ultrasound is the first-line diagnostic modality and demonstrates characteristic bowel wall thickening and the target sign of intussusception. Computed tomography appears to be the most effective and accurate diagnostic modality, identifying 66% of intussusceptions preoperatively with 91% accuracy as compared to ultrasonography, which has a reported accuracy of 60.0% overall.^5^

Management of IgA vasculitis-associated intussusception is primarily conservative, with most cases responding to intensified medical therapy without the need for surgical intervention. Corticosteroids are the cornerstone of treatment for IgA vasculitis, and early initiation may prevent intussusception and intestinal perforation. For corticosteroid-refractory cases, intravenous immunoglobulin has shown benefit while other immunosuppressive agents including dapsone, calcineurin inhibitors, mycophenolate mofetil, and rituximab may be considered. Diaz et al reported the case of a three-year-old girl with IgA vasculitis and ultrasound-confirmed small bowel intussusception who experienced spontaneous resolution of the telescoping segment following fluid resuscitation.^6^

While IgA vasculitis is typically self-limiting, conservative management with close monitoring by an experienced surgical team is feasible for select patients with intussusception, particularly those with ileoileal involvement, confirmed imaging diagnosis, and known time of onset.^7^ Intestinal perforation represents the most serious complication of conservative therapy. Indications for surgical intervention include signs of peritonitis, bowel ischemia or perforation, hemodynamic instability, or failure of conservative management. Cui et al described the case of a 19-year-old male with IgA vasculitis and hematochezia who developed peritonitis and underwent emergent laparotomy, which revealed the ileocecal junction intussuscepting into the ascending colon and a large subserosal hematoma serving as the lead point.^8^ Manual release of the ileocecal intussusception and junction repair were performed, and the patient had no long-term complications from surgery during a six-month follow-up period.^8^ Furthermore, patients with IgA vasculitis-associated intussusception demonstrate higher rates of severe nephritis, underscoring the need for continued monitoring of renal function following ED management.

## CONCLUSION

This case highlights a relatively uncommon presentation of IgA vasculitis-associated intussusception in a 21-year-old male. While IgA vasculitis is primarily a pediatric condition, clinicians must maintain a high index of suspicion in adults presenting with the classic tetrad of palpable purpura, arthralgia, abdominal pain, and renal involvement, particularly when gastrointestinal symptoms are severe. Supportive care with rest, hydration, and analgesics is typically sufficient while glucocorticoids should be considered for severe cases. This case demonstrates that intussusception in the context of IgA vasculitis may be transient and resolve spontaneously; however, surgical intervention should be considered in cases involving signs of peritonitis, bowel ischemia or perforation, hemodynamic instability, or failure of conservative management.
